# Clinical outcomes of sentinel node navigation surgery in patients with preoperatively estimated stage IA endometrial cancer and evaluation of validity for continuing sentinel node navigation surgery based on dispersion of recurrence probability

**DOI:** 10.1007/s10147-023-02449-0

**Published:** 2024-01-05

**Authors:** Tsuyoshi Yamashita, Takahiro Itoh, Takuya Asano, Asuka Suina, Mitsutaka Nishimori, Satoru Munakata, Hideki Satoh

**Affiliations:** 1 Department of Obstetrics and Gynecology, Hakodate Municipal Hospital, 1-10-1 Minato-Cho, Hakodate, Hokkaido 041-8680 Japan; 2 Department of Pathology, Hakodate Municipal Hospital, 1-10-1 Minato-Cho, Hakodate, Hokkaido 041-8680 Japan; 3 Department of Media Architecture, School of Systems Information Science, Future University Hakodate, 116-2 Kamedanakano-Cho, Hakodate, Hokkaido 041-8655 Japan

**Keywords:** Endometrial cancer, Sentinel lymph node, Ultrastaging, Propensity score matching, Validity of continuing sentinel node navigation surgery

## Abstract

**Background:**

To evaluate the feasibility of the use and continuation of sentinel lymph node navigation surgery (SNNS) as an alternative to pelvic lymph node dissection (PLND) for patients with preoperatively estimated stage IA endometrial cancer.

**Methods:**

This retrospective study selected the electronic medical records of all patients who had received CT scans and MRI imaging before surgery from April 1, 2009 to March 31, 2021. Sentinel lymph nodes (SLNs) were detected by administrating ^99m^Tc-phytate and/or indocyanine green into the cervix, and the clinical outcomes of the patients who underwent SNNS or PLND were evaluated. Furthermore, in case of nodal recurrence, a new procedure to determine whether the facility should continue with SNNS or not was developed that compares the maximum likelihood hypothesis and an alternative one based on recurrence rates.

**Results:**

Among 137 patients, SLN biopsies with ultrastaging were performed on 91 patients. The SLN detection rate was 95.6%. Over a 59-month median observation period, no statistically significant differences were shown in overall survival, disease-specific survival and disease-free survival between the SNNS and PLND groups when introducing the propensity score method (p-values: 0.06, 0.153, and 0.625, respectively). Our procedure demonstrated that, in our department without recurrence up to the 65th attempt, it was possible to continue SNNS if a recurrence occurs at the 66th attempt.

**Conclusion:**

This study suggests the validity of SNNS as an alternative to PLND. Even in the absence of evidence from randomized controlled trials, we can confirm the validity of continuing SNNS using our procedure.

## Introduction

Endometrial cancer is the most common gynecologic malignancy in Japan. In 2018, among patients numbering more than 17,000, more than half were diagnosed with early-stage cancer not requiring additional treatments [1]. However, stage IIIC, as diagnosed by pelvic lymph node dissection (PLND), is speculated to have a relatively poor prognosis. For this reason, comprehensive staging surgery including PLND plays an important role in determining postoperative adjuvant therapy. The incidence of PLN metastasis in patients with low risk of recurrence confirmed pathologically is approximately 1.7% to 13% [2, 3]. Since the therapeutic impact of PLND on early-stage patients has yet to be clarified [4–6], it remains controversial whether PLND is necessary for patients with preoperatively estimated stage IA.

Since 2011, sentinel lymph node biopsy (SLNB) for early-stage patients has been used as a diagnostic method for assessing PLN status [7]. Thus, sentinel lymph node navigation surgery (SNNS), in which only one or a few SLNs are removed and further PLND is omitted when metastasis is ruled out intraoperatively, has been suggested as a way to reduce intraoperative surgical damage and postoperative complications such as leg edema and lymph cysts without worsening prognosis as long as certain criteria are met [8–10]. Furthermore, from 2013 to 2014, it was reported that identifying micrometastasis by ultrastaging and offering the opportunity for additional treatment to patients may improve survival compared with that of patients who underwent PLND without ultrastaging [11, 12].

At present, most reports on SNNS, which is a procedure that still does not have national health insurance coverage in Japan, focus on the surgical outcomes of SLNB and the reduction of leg edema and lymph cysts [13–21]. Only one report highlights oncologic outcomes, incorporating three-year overall survival and recurrence-free survival exclusively for the SNNS group [22]. Thus, data comparing the long-term outcomes of SNNS with those of PLND are lacking.

Furthermore, it is essential to maintain high metastatic identification and survival rates when introducing SNNS as an alternative to PLND at each facility. However, caution should be exercised when performing SNNS on patients indicated for PLND in standard clinical practice, as the eligibility and exclusion criteria for randomized controlled trials (RCTs) evaluating SNNS have not yet been reported [23, 24]. Given the low recurrence rate in early-stage patients, there is a risk of continuing SNNS after a recurrence without recognizing the surgical and diagnostic issues.

In this study, we assessed whether SNNS serves as an alternative to PLND by comparing the oncologic outcomes of the SLNB and PLND groups in patients with a preoperatively estimated stage IA. Furthermore, we report a new procedure that compares two different hypotheses to determine whether SNNS should continue if a recurrence occurs before a sufficiently large number of SNNS procedures have been performed [25].

## Patients and methods

### Patients

**Fig. 1:**
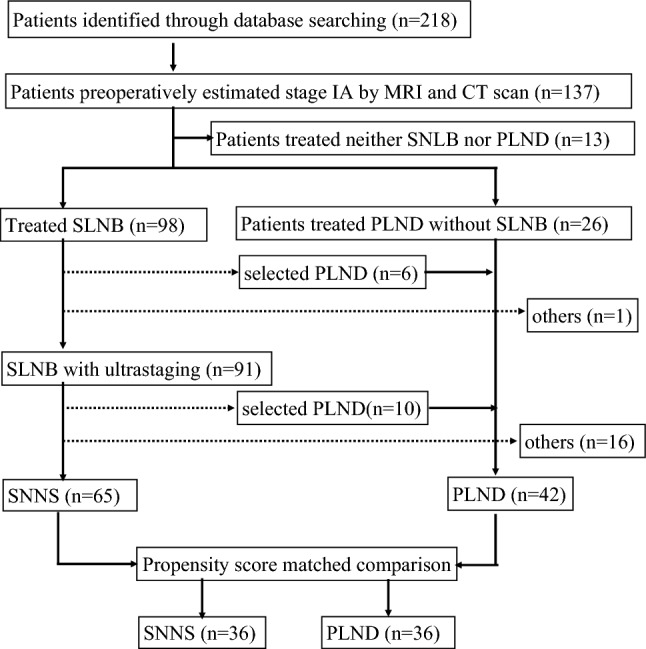
Patient flow chart. SLNB: Sentinel lymph node biopsy PLND: Pelvic lymph node dissection SNNS: Sentinel node navigation surgery (Omission of bilateral pelvic lymph node dissection followed by results of intra-operative frozen section diagnosis of the bilateral SLN)

A consecutive series of patients with endometrial cancer who visited our facility between April 1, 2009, and March 31, 2021, were identified from the hospital`s electronic medical records. All patients were pathologically diagnosed with endometrial cancer, and patients who were preoperatively estimated with stage IA based on preoperative CT scans and MRI imaging were selected for the study. The preoperatively estimated stage IA group was divided into two groups, SLNB or PLND, excluding cases where neither SLNB nor PLND was performed (Fig. 1). The SNNS group was composed of patients who chose to omit further PLND when negative metastasis in SLN was detected intraoperatively and the PLND group was composed of patients who underwent bilateral PLND regardless of whether SLNB was performed.

### Surgical procedures, pathological diagnosis

SLNB procedures for early-stage uterine malignancies have been performed with the approval of our hospital's Institutional Review Board since November 2010, while SNNS procedures began on December 1, 2012, when ultrastaging was introduced. SLNB and SNNS are not standard methods for assessing lymph node metastasis in Japan; patients themselves must make the decision to undergo PLND or SLNB after receiving an explanation of the procedures for those surgeries. If SLNB is chosen, the patient decides whether to undergo SNNS or PLND regardless of the results of the intraoperative diagnosis.

On the day before the surgery, ^99m^Tc-phytate (PDR Pharma, Japan) was injected into the cervix at the 3 o'clock and 9 o'clock positions at a dose of 40 MBq/0.4 ml. Then, lymphoscintigraphy was performed 2 h later to estimate the location of the SLN. On the day of the surgery, 1 ml of indocyanine green (ICG) 0.025 mg/ml (Diagnogreen, Daiichi Sankyo, Japan) was administrated at the 2, 4, 8 and 10 o'clock positions of the cervix, respectively. The SLN was detected using a gamma probe (Navigator GPS, Sheeman Co. Ltd., Japan) and a fluorescent camera (Visera Elite II video system, Olympus, Japan); then it was excised and sent to a pathologist for intraoperative frozen section diagnosis [26, 27]. The diagnostic algorithm of SNNS has as a side-specific PLND: when there is an intraoperative diagnosis of SLN negativity, further removal of PLNs is omitted, and PLND is performed on the positive SLN side or on the side where there is an undetected SLN [8, 9]. Para-aortic lymph node (PAN) dissection was performed with PLND when the patient was preoperatively diagnosed as high-risk and gave consent for PAN dissection, regardless of whether SLNB was performed. An extrafascial extended hysterectomy, a standard hysterectomy in Japan, was performed. Intraoperative diagnosis of cancer metastasis was made by taking frozen section specimens at 2-mm intervals, which were perpendicularly sliced and stained with H&E. After preparing formalin-fixed paraffin embedded tissue (FFPE), the remaining specimens were ultrastaged. Immunohistochemical analysis using cytokeratin AE1/AE3 was performed at 20-μm intervals on a 3-μm FFPE section to detect micrometastasis. A routine histopathological examination was made to evaluate the FIGO stage and risk of recurrence. Low risk was defined as FIGO stage IA endometrioid carcinoma grade 1 or grade 2 with negative lympho-vascular space invasion (LVSI). Intermediate and high risk were defined as any risk other than low risk. For patients postoperatively diagnosed as intermediate or high risk, an additional six cycles of chemotherapy or radiation therapy, and three cycles of chemotherapy if isolated tumor cells (ITC) were confirmed, were proposed and those additional treatments were performed unless refused.

### Statistical analysis

The data obtained were summarized using basic statistics and tested using either a Mann–Whitney test, a chi-square test, or Fisher's exact test. The propensity score method (pair-matching) was utilized to.

adjust for confounding factors in the SNNS and PLND groups. Age (under or over 55), LVSI (negative or positive), histologic subtype (either endometrioid carcinoma grade 1 or 2, or others) and upstaging (FIGO stage IA or others) were used as explanatory variables [28–30]. The propensity score was calculated by logistic regression analysis. The matching caliper was set to 0.2 and it was used to create a 1:1 matched pair from both groups. Then, the survival rates of the created groups were compared using the adjusted Kaplan–Meier curves with a log-rank test. The results were considered significant at p < 0.05 and all tests were two-tailed. All survival analyses were performed using EZR 1.52 (Saitama Medical Center, Jichi Medical University, Saitama, Japan), a GUI of R ver.4.0.0 (The R Foundation for Statistical Computing, Vienna, Austria) [31].

The start of observation was the date of surgery, and the end of observation was either the last hospital visit or death confirmation by September 30, 2021. Overall survival (OS) was calculated from all-cause death events, while disease-specific survival (DSS) was calculated from death events due to recurrence diagnosed by CT scan every six months or from vaginal stump cytology. Disease-free survival (DFS) is defined as the period from the start date to the earlier of either the date of recurrence or the date of death from any cause.

### Development of evaluation procedure for judging whether to continue with SNNS

**Fig.2:**
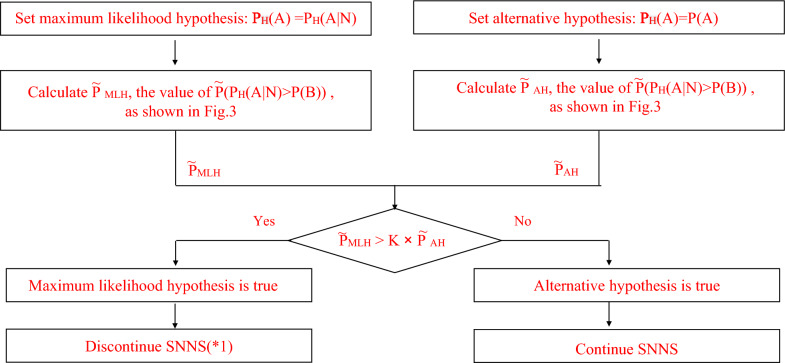
Flow chart to compare maximum likelihood hypothesis and alternative hypothesis (*1). The maximum likelihood hypothesis means P_H_(A) = P_H_(A/N) and this procedure is done when P_H_(A|N) > P(B). Thus, P_H_(A) > P(B) and SNNS should be discontinued

The nodal recurrence rate after SNNS, P(A), is 1.2% and the nodal recurrence rate after PLND, P(B), is 1.7% in a systematic review [32]. Consider a facility H where P_H_(A), the true recurrence rate after SNNS, is 1.2%. It is obvious that SNNS should be performed if they know the value of P_H_(A). However, they have no way of knowing it. The only performance index that they can use to judge whether to continue SNNS or not is thus P_H_(A|N), the recurrence rate obtained from N SNNSs (the number of SNNSs) performed at the facility H. However, in the case of P_H_(A) = 1.2% and P(B) = 1.7%, P(P_H_(A|N) > P(B)), the probability that P_H_(A|N) is greater than P(B), is 50.4% when N = 58 if recurrences occur randomly with probability P_H_(A). Therefore, it is difficult for the facility to make a judgement on the basis of P_H_(A|N) about whether they should continue performing SNNS if P_H_(A|N) > P(B) at the beginning of SNNS. We thus developed a new procedure to solve this problem when P_H_(A|N) > P(B), as shown in Fig. 2. Here, K is a sufficiently large value greater than one: K = 2 in this study. Note that it is obvious that SNNS should be continued regardless of the value of $\tilde{{\text{P}}}$(P_H_(A|N) > P(B)), an estimate of P(P_H_(A|N) > P(B)), when P_H_(A|N) ≦ P(B).

**Fig. 3:**
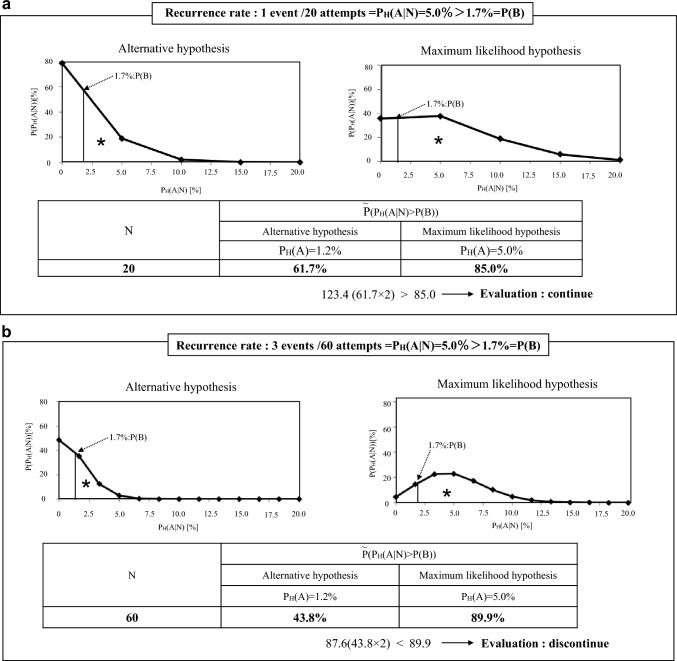
$\tilde{{\text{P}}}$(P_H_(A|N) > P(B)) [%] (P(B) = 1.7% for maximum likelihood hypotheses and alternative hypothesis. a: at 1 event per 20 attempts, b: at 3 events per 60 attempts. P(B): Recurrence rate after PLND (1.7%) obtained from a systematic review. P_H_(A): True recurrence rate after SNNS in facility H, which it is impossible to know in advance. n_A_: The number of recurrences in N SNNSs (a random variable). ◆: A binomial distribution B (N, P_H_ (A)) that obeys n_A._ P_H_(A|N) = n_A_/N: Recurrence rate after SNNS obtained from N SNNSs performed in facility H, which is a random variable because n_A_ is a random variable. ─◆─: Probability density function for P_H_(A|N), which is an approximation of B (N, P_H_ (A)) when P_H_ (A|N) is treated as a continuous random variable. *: Area under curve (AUC) based on the probability density function of each hypothesis. For instance, 61.7 for the alternative hypothesis in 20 attempts means the percentage to the right of 0.017 when the overall area of the graph is set to 1. $\tilde{{\text{P}}}$(P_H_(A|N) > P(B)): The areas indicated by * in the four graphs, which means an estimate of the probability that P_H_ (A|N) is greater than P(B)

Consider an example where N = 20 and n_A_ = 1 (P_H_(A|N) = 5.0%, Fig. 3a). According to the procedure described above, SNNS should be continued in this case, although P_H_(A|N) = 5.0% is sufficiently greater than P(B) = 1.7%. This is because $\tilde{{\text{P}}}$(P_H_(A|N) > P(B)) for the maximum likelihood hypothesis is 85.0% and is less than two times that for the alternative hypothesis (61.7%) (Table 1); that is, P_H_(A) > P(B) is insignificant (the alternative hypothesis is significant) according to the procedure with K = 2. However, if n_A_ = 3 for N = 60 (see Fig. 3b), SNNS should be discontinued although P_H_(A|N) = 5.0%, in the same manner as in the above case with N = 20. This is because $\tilde{{\text{P}}}$(P_H_(A|N) > P(B)) for the maximum likelihood hypothesis is 89.9% and is greater than two times that for the alternative (43.8%) (Table 1); that is, P_H_(A) > P(B) is significant (the maximum likelihood hypothesis is significant). We can thus evaluate the validity of performing SNNSs in an actual situation where N is less than 100 by using a procedure with a criterion that is intuitively easy to understand.

**Table 1:** $\tilde{{\text{P}}}$(P_H_(A|N) > P(B)): [%](P(B) = 1.7%) for maximum likelihood hypotheses and alternative hypothesis at each attempt

N	$\tilde{{\text{P}}}$(P_H_(A|N)^1^ > P(B)^2^)^3^%				
	Alternative hypothesis	Maximum likelihood hypothesis			
	P_H_(A) ^4^= 1.2%	P_H_(A) = 2.0%	P_H_(A) = 3.0%	P_H_(A) = 5.0%	P_H_(A) = 8.0%
20	61.7	69.4	76.3	85.0	92.0
30	54.4	65.5	74.9	85.9	93.9
40	49.9	63.4	74.5	87.2	95.5
45	48.2	62.6	74.4	87.8	96.2
57	44.7	60.7	74.4	89.5	97.6
60	43.8	60.3	74.4	89.9	97.8
83	38.1	58.3	75.9	93.0	99.1
155	29.2	58.3	82.5	89.0	100.0
…	…	…	…	…	…
1000	8.1	74.3	99.5	100.0	100.0

^1^ P_H_(A|N): Recurrence rate after SNNS obtained from N SNNSs performed in facility H (random variable)

^2^ P(B): Recurrence rate after PLND (1.7%) obtained from a systematic review

^3^ $\tilde{{\text{P}}}$(P_H_(A|N) > P(B)): Estimate of the probability that P_H_(A|N) > P(B)

^4^ P_H_(A): True recurrence rate by SNNS in facility H, which it is impossible to know in advance

The values of $\tilde{{\text{P}}}$(P_H_(A|N) > P(B)) are shown for the maximum likelihood hypotheses with P_H_(A) = 2.0%, 3.0%, 5.0%, and 8.0% and the alternative one with P_H_(A) = 1.2%. Here, some of the values of N have been selected on account of space considerations so that $\tilde{{\text{P}}}$(P_H_(A|N) > P(B)) for the maximum likelihood hypothesis is equal to around two times that for the alternative (see the cells of N = 45, 57, 83, and 155 with gray background, which are the minimum value of N at which we can judge P_H_(A) > P(B)). Those at N = 20,30,40,60, and 1000 were also selected to show how $\tilde{{\text{P}}}$(P_H_(A|N) > P(B)) converges. The table indicates that the number of cases necessary to judge P_H_(A) > P(B) decreases as P_H_(A) becomes large

## Results

### Surgical and pathological outcomes

A total of 218 consecutive pathologically confirmed patients were selected from the hospital`s electronic medical records, and 91 out of the 137 patients with preoperatively estimated stage IA underwent SNLB with ultrastaging. The patients’ characteristics and clinical results are shown in Table 2. Of those with preoperative estimated stage IA, 76.9% were FIGO stage IA, 23.1% were upstaged to stage IB or higher, and the rate of the histologic type of endometrioid carcinoma grade 1 or 2 was 82.4%. The overall detection rate and bilateral detection rate of SLN were 95.6% and 78.0%, respectively. The detection rates with ^99m^Tc-phytate and/or ICG were 90% or more and there was no difference in detection rate between two tracers (p = 0.57). Metastasis and ITC to SLN were identified in 9.9% and 6.6% of the patients, respectively. No surgical complications were found to have occurred during SNLB.

**Table 2:** Characteristics and surgical results of patients with SLNB followed by postoperative ultrastaging

Age (years)		58.1 (median)
Surgical approach		
	Laparotomy	12 (13.2%)
	Laparoscopy	79 (86.8%)
	Para aortic lymph-node dissection	1 (1.1%)
FIGO stage		
	IA	70 (76.9%)
	≧IB	21 (23.1%)
Histology		
	Endometrioid (G1or G2)	75 (82.4%)
	Other than Endometrioid (G1 or G2)	16 (17.6%)
Number of SNL		2.7(mean)
SNL detection rate		
	Bilateral	71 (78.0%)
	Hemi lateral	16 (17.6%)
	Non detected	4 (4.4%)
SNL detection rate by tracer		
	^99m^Tc-phytate	93.3% (83/89)
	ICG	90.4% (66/73)
SNL status		
	Isolated tumor cell	6 (6.6%)
	micrometastasis	6 (6.6%)
	macrometastasis	3 (3.3%)
PLND (pelvic lymph-node dissection)		
	Bilateral PLND	10
	Side specific dissection	12
	Bilateral omission	69
Upstage (≧IB)		21(23.1%)
		(n = 91)

### Survival analysis

**Fig. 4:**
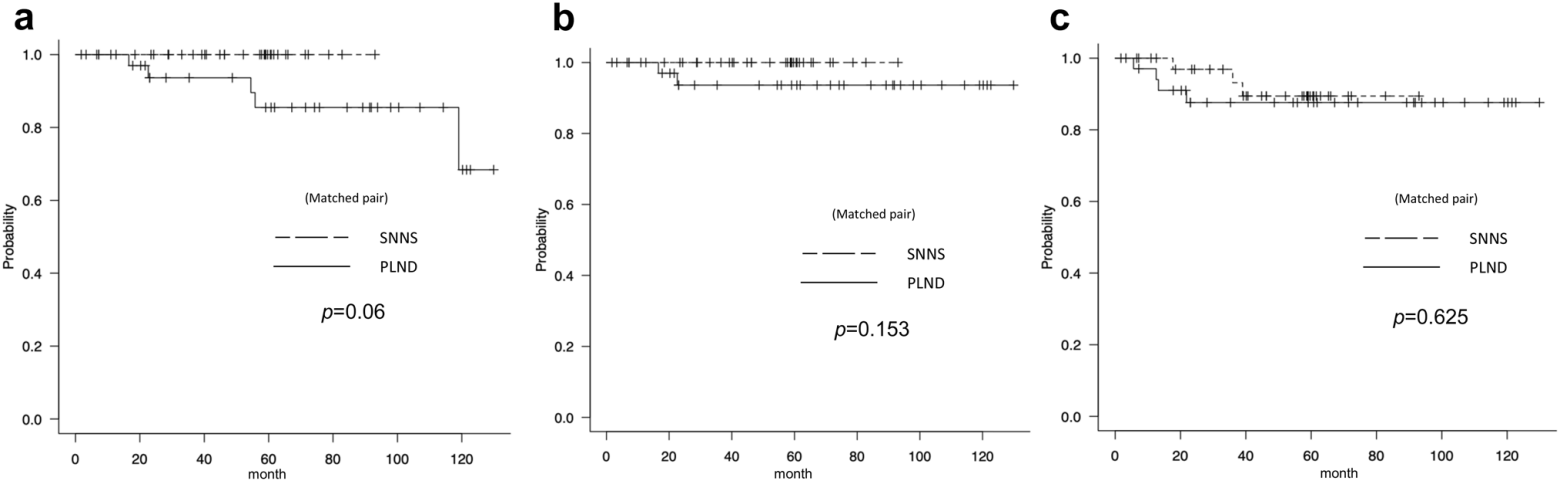
Survival outcomes of SNNS and PLND groups determined by the propensity score matching method** a**: Overall survival adjusted by PS,** b**: Disease specific survival adjusted by PS,** c**: Disease-free survival adjusted by PS

Of the 137 patients, 65 underwent SNNS and 42 underwent PLND. The median observation time for the 107 patients in the SNNS and PLND groups was 59 months. The results of the survival analysis are shown in Fig. 4 and Table 3. There were significant differences in pathological type and LVSI ratio between the two groups (p = 0.01 and p = 0.026, respectively), so the propensity score method was used to adjust for confounding factors. As a result, log-rank tests of adjusted OS, DSS, and DFS showed p-values of 0.06, 0.153 and 0.625, respectively, indicating no significant difference between the two groups. The five-year survival rates were 100% and 85.5% for SNNS and PLND, respectively, and the five-year DSS rates were 100% and 93.6%.

**Table 3:** Comparison of clinical and pathological data between SNNS and PLND groups after introduction of propensity score matching method

						Matched pair by propensity score method			
		SNNS(n = 65)	PLND(n = 42)	*p* value	*SMD	SNNS(n = 36)	PLND(n = 36)	*p* value	*SMD
Age (years)				0.156	0.316			1	0.057
	< 55	30(46.1%)	13(31.0%)			14(38.9%)	13(36.1%)		
	≧55	35(53.9%)	29(69.0%)			22(61.1%)	23(63.9%)		
Surgical characteristics									
	Surgical time (min.)	187	230	0.008		180	228	0.02	
	Bleeding (g)	52	186	< 0.001		54	184	< 0.001	
FIGO stage									
	IA	52 (80.0%)	31(73.8%)	0.484		27(75.0%)	26(72.2%)	1	
	≧IB	13(20.0%)	11 (26.2%)			8(25.0%)	9(27.2%)		
Histology				0.01	0.524			1	< 0.001
	Endometrioid (EM) G1or G2	56(86.2%)	27 (62.3%)			27(75.0%)	27(75.0%)		
	Other than EM G1 or G2	9(13.8%)	15 (35.7%)			9(25.0%)	9(25.0%)		
LVSI (lymph-vascular space invasion)				0.026	0.293			1	0.061
	Positive	12(18.5%)	13(31.0%)			11(30.6%)	10(27.8%)		
	Negative	53(81.5%)	19(45.2%)			25(69.4%)	26(62.2%)		
Upstage		11	13	0.102	0.147	10	9	1	0.063
Overall survival (OS)									
	3Y(95%CI)	100%	89.1% (73.5–95.8)			100%	93.6% (76.8–98.4)		
	5Y(95%CI)	100%	82.5% (64.9–91.8)	0.0004		100%	85.5% (65.5–94.4)	0.06	
Disease specific survival (DSS)									
	1Y(95%CI)	100%	89.1% (73.5–95.8)			100%	93.6% (76.8–98.4)		
	3Y(95%CI)	100%	89.1% (73.5–95.8)	0.0126		100%	93.6% (76.8–98.4)	0.153	
Disease free survival (DFS)									
	3Y(95%CI)	100%	92.4% (78.3–97.5)			100%	97.1% (80.9–99.6)		
	5Y(95%CI)	94.2% (83.0–98.1)	84.5% (68.6–92.7)	0.275		93.1% (75.1–98.2)	87.6% (70.2–95.2)	0.625	
Number of death (overall)		0	7			0	5		
Number of death (disease specific)		0	4			0	2		
Number of recurrences		5	6			3	4		
Site of recurrence	pelvic cavity	0	1			–	–		
	other than pelvic cavity	5	6			–	–		

^*^SMD Standardized mean difference

### Evaluation of procedure for judging whether to continue with SNNS

We discussed the advisability of continuing SNNS attempts in our department, which has had 65 SNNS attempts, by using the procedure developed in this study. As a result, it was found that it is possible to continue SNNS if a recurrence occurs at the 66th attempt. This is because there was no recurrence up to the 65th attempt in our department and thus $\tilde{{\text{P}}}$(P_H_(A|N) > P(B)) was less than the given criterion (Table 1).

## Discussion

In 2008, Marian et al. proposed an option to omit PLND for early-stage endometrial cancer patients with a low risk of recurrence under certain conditions. However, this approach had an issue of low specificity despite its high sensitivity in detecting PLN metastasis [33, 34]. When SNNS is introduced in each facility as an alternative to PLND, it is important to maintain metastatic identification and survival rates that are at least comparable to those of PLND while ensuring a high detection rate for SLN. So far, methods using ICG and a fluorescence camera have been reported to achieve sufficiently high SLN detection rates [35, 36]. Moreover, in seven recent studies using ^99m^Tc, the detection rates ranged from 91–98% (median 93.8%) overall, and 63–88% (median 79.2%) for bilateral findings [19, 37–42]. Additionally, a significant amount of data from the Japan Society of Gynecology and Obstetrics indicates the five-year survival rate for stage IA patients in Japan at around 96% [1]. If these conditions are met, SNNS could potentially replace PLND as a less invasive procedure.

This is the first report on oncologic outcomes, such as OS and DSS, for the SNNS and PLND groups of Japanese patients with a preoperatively estimated stage IA endometrial cancer. In this study, the detection rates of SLN and SLN metastases were comparable to previous reports, and there was no statistically significant difference in adjusted OS using the propensity score method between the SNNS and PLND groups. Furthermore, DSS and DFS were calculated as additional indicators for clinical judgment, and it was concluded that there was no significant difference in any of the survival outcomes between the two groups. Additionally, the five-year survival rate of patients who underwent SNNS at our institution appeared to align with data from a large Japanese database. Although SNNS in this study seems to be a promising alternative diagnostic approach for detecting PLN metastasis and reducing surgical damage, we must ultimately await the results of reliable RCTs with a larger sample size to determine if SNNS is a suitable diagnostic alternative to PLND.

Lymph node recurrence rates, which may be related to survival rates, can also serve as an indicator of the validity of SNNS. Generally, due to the learning curve, surgeons typically require 30 to 40 procedures to perform SNNS reliably [43–45]. When a nodal recurrence is significantly more frequent with SNNS than with PLND, SNNS should be discontinued. However, for instance, if a nodal recurrence occurs in fewer than 100 attempts, especially at the beginning of SNNS, it might be difficult to distinguish between a random error, where recurrence might occur even with PLND, and a systematic error from SNNS surgical technique. To solve this problem, we need a statistical test to judge whether SNNS should be continued or not. Also, we may need a statistical matching technique such as propensity score matching to reduce the bias due to the effect of a treatment or other intervention. However, we cannot always use these conventional techniques. This is because P(A) and P(B) are too small (1.2% and 1.7%) for the above situation where a sufficiently large number of SNNSs have not been performed at each facility at the beginning of SNNS, and the conventional techniques mentioned above need a sufficiently large number of attempts to evaluate the results of SNNS with high accuracy. We thus developed a new procedure that involves comparing the maximum likelihood hypothesis, where P_H_(A) = P_H_(A|N), with the alternative, where P_H_(A) = 1.2%. The procedure can be used at the beginning of SNNS when there have been fewer than 100 attempts and it shows us whether P_H_(A|N) is within an allowable dispersion or not. We can thus stop performing SNNS if P_H_(A|N) is beyond the allowable dispersion and can investigate the reason why it is high. This enables us to decrease the chances that patients suffer from selecting the wrong operative method. As mentioned before, P_H_(A|N) is a random variable; $\tilde{{\text{P}}}$(P_H_(A|N) > P(B)) thus contains a stochastic dispersion of up to two times when recurrences occur randomly. Therefore, we set K = 2 in our study. However, recurrences do not always occur randomly. Thus, it is necessary to investigate the recurrence course and establish a method to set the value of K.

In conclusion, SNNS with cervical injection of tracers appears to be a safe alternative to PLND without increasing recurrences or decreasing survival rates, provided the SLN detection rate remains high and micrometastasis is identified through ultrastaging. However, as evaluations of SNNS using RCTs have not yet been reported, caution should be exercised in continuing SNNS, especially in the event of a nodal recurrence. The decision to proceed with SNNS should be based on verifiable and appropriate criteria. In order for SNNS to be covered by national health insurance in Japan, it is necessary to increase the number of cases or the number of facilities performing SNNS and to demonstrate its efficacy as an alternative to PLND. Additionally, the issues due to financial or human resources, such as the adoption of OSNA (one-step nucleic acid amplification) assays and pathological ultrastaging methods for detecting SLN metastasis, remain to be addressed in the future.
